# The E3 ubiquitin ligase RNF121 is a positive regulator of NF-κB activation

**DOI:** 10.1186/s12964-014-0072-8

**Published:** 2014-11-12

**Authors:** Naima Zemirli, Marie Pourcelot, Neslihan Dogan, Aimé Vazquez, Damien Arnoult

**Affiliations:** INSERM, UMR_S 1014, Hôpital Paul Brousse, Villejuif, 94800 France; Université Paris-Sud P11, Orsay, 91400 France; Equipe Labellisée Ligue contre le Cancer, Villejuif, 94800 France

**Keywords:** NF-κB, Ubiquitination, E3 ubiquitin ligase, Golgi apparatus, Innate immunity

## Abstract

**Background:**

The nuclear factor κB (NF-κB) family members regulate several biological processes as cell proliferation and differentiation, inflammation, immunity and tumor progression. Ubiquitination plays a key role in NF-κB activation and the ubiquitylated transmitters of the NF-κB signaling cascade accumulate in close proximity to endomembranes.

**Findings:**

We performed an unbiased siRNA library screen targeting the 46 E3 ubiquitin ligases bearing transmembrane domains to uncover new modulators of NF-κB activation, using tumor necrosis factor–α (TNF-α) receptor (TNFR) stimulation as a model. We report here the identification of a new Golgi Apparatus-resident protein, RNF121, as an enhancer of NF-κB promoter activity through the catalytic function of its RING domain. From a molecular standpoint, while knocking down RNF121 did not alter RIP1 ubiquitination and IKK activation, the proteasomal degradation of IκBα was impaired suggesting that this E3 ubiquitin ligase regulates this process. However, RNF121 did not directly ubiquitinate IκBα While they were found in the same complex. Finally, we discovered that RNF121 acts as a broad regulator of NF-κB signaling since its silencing also dampens NF-κB activation following stimulation of Toll-Like Receptors (TLRs), Nod-Like Receptors (NLRs), RIG-I-Like Receptors (RLRs) or after DNA damages.

**Conclusions:**

These results unveil an unexpected role of Golgi Apparatus and reveal RNF121 as a new player involved in the signaling leading to NF-κB activation.

**Electronic supplementary material:**

The online version of this article (doi:10.1186/s12964-014-0072-8) contains supplementary material, which is available to authorized users.

## Findings

The transcription factor NF-κB plays pivotal roles in the regulation of a plethora biological processes, including cell proliferation and differentiation, innate and adaptive immunity, inflammation and tumor progression [1,2]. NF-κB is a homo or heterodimer constituted with subunits belonging to the Rel family and the NF-κB dimers are sequestered in the cytosol by a member of the Inhibitor of κB (Iκb) family [2,3].

NF-κB is activated by many inducers. Each inducer is recognized by a receptor at the cell surface or within the cell, and its binding triggers a specific signaling pathway leading to NF-κB activation [2,3]. Each pathway involves the organization of signaling protein complexes, the formation of polyubiquitin chains acting as binding agents on specific transmitters and the action of kinases [4,5]. Nevertheless, all these complexes have a point in common: they recruit and activate, through specific ubiquitylated transmitters, the inhibitor of NF-κB (IκB) kinase (IKK) complex, which consists of two catalytic kinases, IKKα and IKKβ, and the regulatory subunit NEMO (also known as IKKγ) [6]. IKK then phosphorylates the inhibitory IκB proteins, promoting their Lys^48^ (K^48^)–linked ubiquitination and proteasomal degradation [7]. NF-κB dimers subsequently enter the nucleus, where they initiate the transcription of their target genes, including genes encoding pro-inflammatory cytokines or anti-apoptotic proteins [1].

**Figure 1:**
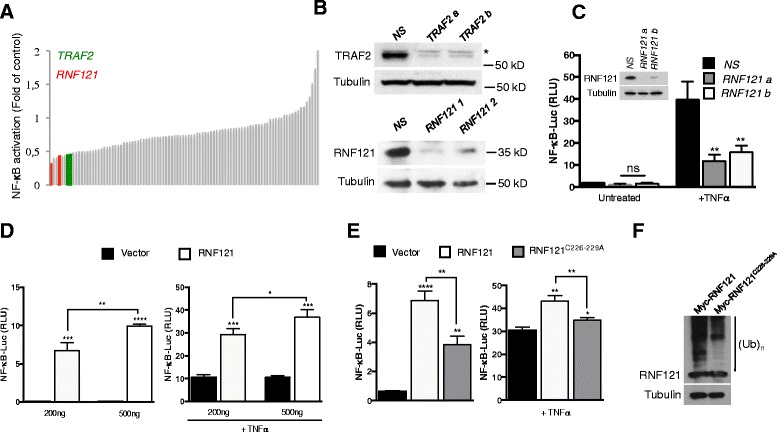
**Participation of RNF121 in TNFR-mediated NF-κB activation. (A)** NF-κB reporter luciferase assay screen of a siRNA library targeting 46 transmembrane E3 ubiquitin ligases (2 siRNAs/target) in HEK293T cells. Cells were stimulated with TNFα (10 ng/ml) for 6 hrs and fold activation compared to non-specific (*NS*) siRNA-treated cells was calculated. Red and green histograms indicate siRNA against RNF121 and TRAF2, respectively. TRAF2 was used as a positive control. **(B)** Cell extracts from HEK293T cells transfected as in **(A)** were analyzed by immunoblot as indicated. **(C)** HEK293T cells transfected with a control non-specific (*NS*) siRNA or with siRNAs against RNF121 (*RNF121 a* or *b*), were also transfected 48 hrs later with an NF-κB reporter. 24 hrs later, the cells were either left unstimulated or were stimulated with TNFα (10 ng/ml) for 6 hrs and then were analyzed by luciferase assay. The results were normalized against *Renilla* luciferase activity [analysis of variance (ANOVA)]. ns: not significant. RLU, Relative Light Units. Inset: Immunoblotting analysis of the knockdown of RNF121 by the specific siRNAs. **(D)** NF-κB reporter luciferase assay in HEK293T cells transfected with increasing concentrations (200 or 500 ng) of a Myc-tagged plasmid coding for RNF121 and left unstimulated (left panel) or stimulated with TNFα (1 ng/ml) for 6 hrs (right panel) [analysis of variance (Student’s *t*-tests)]. **(E)** NF-κB reporter luciferase assay in HEK293T cells transfected with 200 ng of a Myc-tagged plasmid coding for RNF121 or for the mutant RNF121^C226-229A^ and left unstimulated (left panel) or stimulated with TNFα (10 ng/ml) for 6 hrs (right panel) [analysis of variance (Student’s *t*-tests)]. **(F)** HEK293T cells were transfected with a Myc-tagged plasmid coding for RNF121 or for the mutant RNF121^C226-229A^. 24 hrs later, cell extracts were analyzed by immunoblotting. (Ub)_n_ indicates poly-ubiquitylated species.

As we recently reported an accumulation of the ubiquitylated transmitters leading to NF-κB activation to the endomembrane fraction [8], we set up a siRNA screen with two oligoribonucleotides against each of the 46 membrane spanning E3 ubiquitin ligases [9] (Additional file 1) to uncover new regulators of NF-κB activation, using tumor necrosis factor–α (TNF-α) receptor (TNFR) stimulation as a model. Indeed, engagement of TNFR promotes a rapid NF-κB activation through the recruitment to the receptor of the adaptor protein TRADD together with the E3 ubiquitin ligases c-IAPs and TRAF2, which are responsible for catalyzing the polyubiquitination of the kinase RIP1 that acts as a specific ubiquitylated transmitter for this pathway [10]. The impact of the knock down of the 46 membrane spanning E3 ubiquitin ligases was assessed in a gene reported assay and silencing of the key regulator of TNFR-mediated NF-κB TRAF2 was used as a control (Figure 1A, B; see Additional file 2 for detailed Methods description). Among the top hits was RNF121 (Figure 1A). RNF121 is part of a chromosomal band (11q13) that may contain a high penetrance gene for breast cancer [11]. We then used two additional siRNA sequences against RNF121 and confirmed that TNFR-mediated NF-κB activation was decreased, further validating the results from our initial screen (Figure 1C). Interestingly, the enforced expression of RNF121 activated NF-κB and NF-κB activation following TNFR stimulation was potentiated in a dose dependent-manner (Figure 1D). RNF121 specifically triggered NF-κB activation because it did not stimulate the expression of IFNβ-, ISRE-, NFAT-, AP1- or p53-dependent reporter genes (data not shown). The ability of RNF121 to activate NF-κB was dependent on the catalytic activity of its RING domain, because the RNF121^C226-229A^ mutant gave significantly lower levels of NF-κB activation (Figure 1E). Based on the available RING finger protein structure [12,13], the replacement of the cys-226 and 229 residues with an alanine was predicted to prevent Zn^2+^ coordination, thereby impeding the overall function of the RING domain as assessed by the level of auto-ubiquitination (Figure 1F).

**Figure 2:**
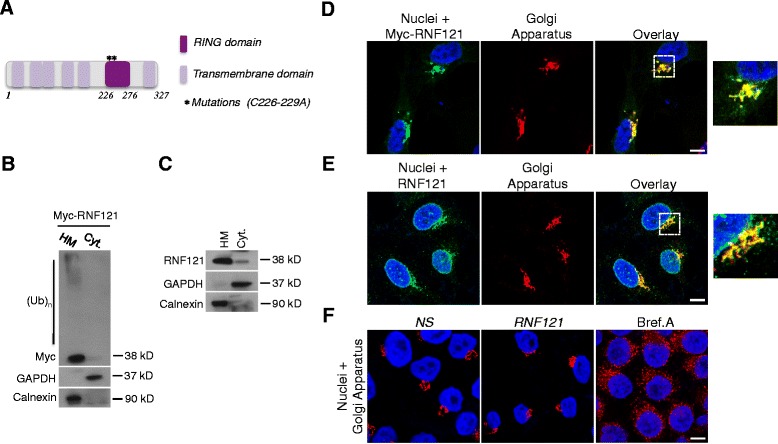
**RNF121 is a Golgi Apparatus-anchored E3 ubiquitin ligase. (A)** Scheme showing the main domains of RNF121. The mutations used in Figure 1E and 1F are also indicated. **(B)** HEK293T cells were transfected with a Myc-tagged plasmid coding for RNF121. 24 hrs later, crude heavy membranes (HM) and cytosolic (Cyt.) fractions were analyzed by immunoblotting as indicated. Calnexin and GAPDH served as purity controls for the HM or cytosolic fraction respectively. (Ub)_n_ indicates poly-ubiquitylated species. **(C)** Crude heavy membranes (HM) and cytosolic (Cyt.) fractions from HEK293T were analyzed by immunoblotting as indicated. **(D)** HeLa cells were transfected with a Myc-tagged plasmid coding for RNF121. 24 hrs later, the localization of myc-RNF121 was investigated by immunofluorescence. GM130 was used as a marker for the Golgi Apparatus. Nuclei were stained with DAPI. Representative images are shown, with the boxed areas enlarged on the right. **(E)** Analysis of the localization of endogenous RNF121 in HeLa cells by immunofluorescence. **(F)** HeLa were transfected with a control nonspecific siRNA or with a siRNA raised against RNF121. 72 hrs later the Golgi Apparatus morphology was examined. As a control, HeLa cells were treated with Brefeldin A (Bref. A) (50 ng/ml) for 3 hrs. Scale bar : 20 μm.

RNF121 is a 327 amino acids protein with a RING domain, and six transmembrane domains that have reported to anchor the protein at the endoplasmic reticulum (ER) in the nematode [14] (Figure 2A). Accordingly, both ectopically expressed and endogenous RNF121 were detected in fractions enriched with intracellular organelles (Figure 2B and C). Next, analysis of the cellular localization of RNF121 by immunofluorescence showed that Myc-tagged or endogenous RNF121 specifically co-localized with the Golgi Apparatus (Figure 2D and E). Finally, while RNF121 appeared to be anchored in the Golgi Apparatus, silencing of this protein did not seem to affect the morphology of this organelle unlike a Brefeldin A treatment (Figure 2F), and the cell viability was not significantly modified (data not shown).

**Figure 3:**
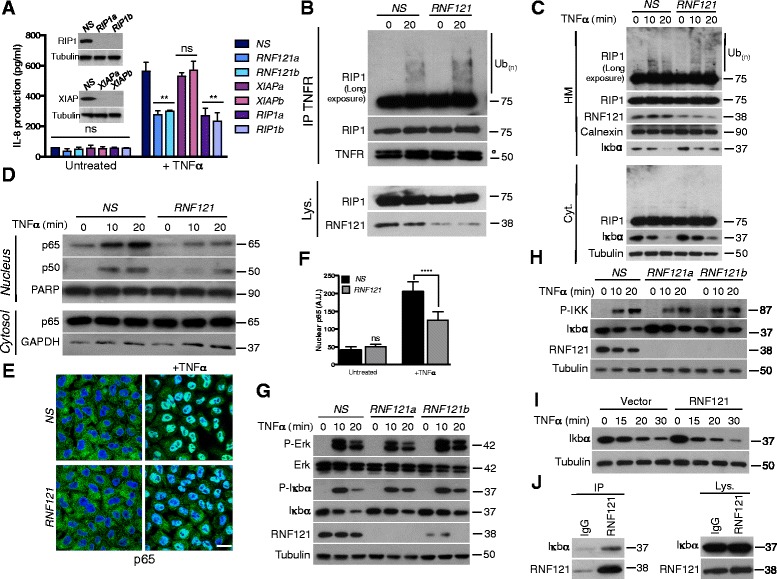
**RNF121 regulates NF-κB activation through IκBα degradation. (A)** HEK293T cells were transfected with a control non-specific *(NS)* siRNA or with the indicated siRNAs. 72 hrs later, the cells were either left untreated or exposed to TNFα (1 ng/ml). The secretion of IL-8 was measured by ELISA. **(B)** HEK293T cells were transfected for 72 hrs with *NS* siRNA or with a siRNA against RNF121 (*RNF121*). Cells were then either left untreated or exposed to 10 ng/ml TNFα for 20 min. Cell lysates (Lys.) were subjected to immunoprecipitation (IP) with an antibody raised against TNFR. ° IgG heavy chains. **(C)** NS- and RNF121-silenced HEK293T cells were stimulated with TNFα (10 ng/ml) for the indicated times. Crude HM and cytosolic (Cyt.) fractions were analyzed by immunoblotting. **(D)** Nuclear and cytoplasmic extracts from cells stimulated as in **(C)** were analyzed by immunoblotting as indicated. **(E)** NS- and RNF121-silenced HeLa cells were either left untreated or exposed to TNFα for 20 min. Nuclear translocation of the p65 NF-κB subunit was assessed by immunofluorescence. Scale bar : 50 μm. The pixel intensity of the nuclear signal of p65 in each condition was quantified in **(F)**. A.U.: Arbitrary unit. **(G)** NS- and RNF121-silenced HEK293T were stimulated with TNFα for the indicated times, then were subjected to immunoblotting analysis as indicated. **(H)** Same conditions as **(G)** but cells were pre-treated 5 min with the broad phosphatase inhibitor Calyculin A (50 nM) before TNFα stimulation. **(I)** HEK293T cells were transfected with a control empty vector or Myc-tagged plasmid coding for RNF121. 24 hrs later, cells were stimulated with TNFα for the indicated times and cell extracts were analyzed by immunoblotting as indicated. **(J)** Cell lysates (Lys.) from HEK293T were subjected to immunoprecipitation (IP) with an antibody raised against RNF121 or with a control IgG.

While RNF121 and RNF175 are close homologs (Additional file 3A), silencing of RNF175 had no overt effect on TNFR-mediated NF-κB activation (Additional file 3B) suggesting a specificity of RNF121 in this pathway. Next, as for RIP1, RNF121 silencing impaired the secretion of the cytokine IL-8, a target gene of NF-κB [1], further confirming the participation of RNF121 in TNFR-mediated NF-κB activation (Figure 3A). RNF121 silencing also sensitized cells to TNFα-mediated cell death (Additional file 4) confirming again the involvement of RNF121 in NF-κB activation as NF-κB protects from TNFα-mediated cell death through the expression of anti-apoptotic proteins [1]. Because ubiquitination of RIP1 is the central event in TNFR signaling [4,5,10], we next investigated whether RNF121 silencing impacts this process. Immunoprecipitation of the TNFR indicated that RIP1 ubiquitination is not altered when RNF121 was knocked down (Figure 3B) and the accumulation of ubiquitylated RIP1 in the heavy membrane fraction was not impeded [8] (Figure 3C). Unexpectedly, RIP1 deubiquitination in RNF121-silenced cells seemed to be delayed (Figure 3C). Nevertheless and as expected, cell fractionation experiments showed reduced p65/p50 NF-κB dimer levels in nuclear fractions from RNF121-silenced cells (Figure 3D) and the decreased relocation of p65 into the nuclei was confirmed by confocal microscopy (Figure 3E and F). These observations were in agreement with the finding that the proteasomal degradation of IκBα was hampered (Figure 3C, G, H and Additional file 5). Nevertheless, as for the MAPKs extracellular signal-regulated kinase (ERK), IκBα phosphorylation was not inhibited when RNF121 was knocked down (Figure 3G) in accord with the observation that activation of the IKK complex was not reduced either (Figure 3H). Together, these results suggested that RNF121 is likely implicated in the control of the proteasomal degradation of IκBα. Accordingly, ectopic expression of RNF121 accelerated IκBα degradation following TNFα exposure (Figure 3I). Immunoprecipitation of endogenous RNF121 demonstrated that both proteins are in the same complex (Figure 3J) and a pool of IκBα co-localized with RNF121 (Additional file 6). However, RNF121 did not seem to induce the ubiquitination of IκBα (Additional file 7A and B) likely because both proteins did not directly interact (Additional file 7A and C).

**Figure 4:**
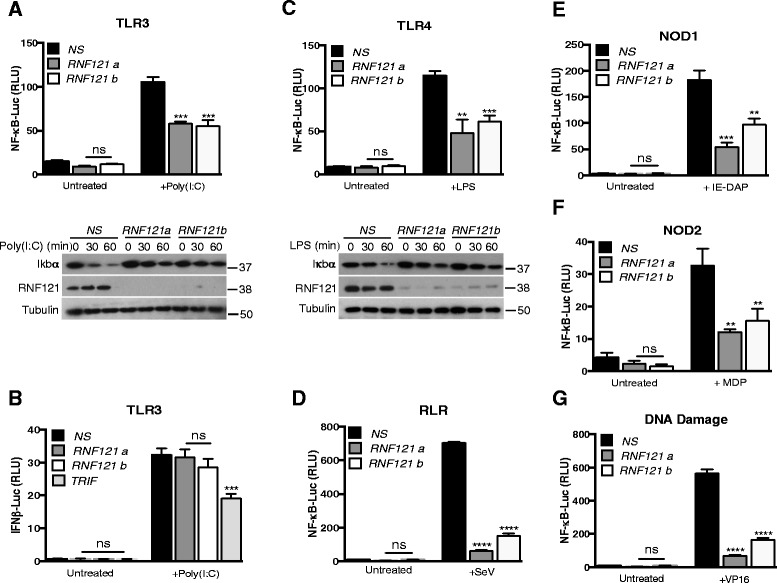
**RNF121 is a broad regulator of NF-κB activation. (A)** HEK293T cells stably expressing TLR3 were transfected with a control non-specific (*NS*) siRNA or with siRNAs against RNF121 (*RNF121 a* or *b*). 48 hrs later, the cells were also transfected with an NF-κB reporter. 24 hrs later, the cells were either left unstimulated or were stimulated with poly(I:C) (10 μg/ml) for 6 hrs and then were analyzed by luciferase assay with normalization against *Renilla* luciferase activity. The NS- and RNF121-silenced cells were also stimulated with poly(I:C) for the indicated times, then were subjected to immunoblotting analysis as indicated. **(B)** NS-,RNF121- and TRIF-silenced HEK293T stably expressing TL3 were transfected with an IFNβ reporter. 24 hrs later, the cells were either left unstimulated or were stimulated with poly(I:C) (10 μg/ml) for 6 hrs and then were analyzed by luciferase assay. **(C)** HEK293T cells stably expressing TLR4 were transfected as in **(A)**. Then, cells were either left unstimulated or were stimulated with LPS (10 μg/ml) for 6 hrs and then were analyzed by luciferase assay. The NS- and RNF121-silenced cells were also stimulated with LPS (10 μg/ml) for the indicated times, then were subjected to immunoblotting analysis as indicated. **(D)** HEK293T cells were transfected as in **(A)**. Then, cells were either left unstimulated or were infected with Sendai virus (SeV) for 6 hrs and then were analyzed by luciferase assay. HEK293T cells stably expressing NOD1 **(E)** or NOD2 **(F)** were transfected as in **(A)**. Then, cells were either left unstimulated or were stimulated with IE-DAP or MDP (10 μg/ml) respectively for 6 hrs and then were analyzed by luciferase assay. **(G)** HEK293T cells were transfected as in **(A)**. Then, cells were either left unstimulated or were treated with etoposide (VP16, 40 μM) for 6 hrs and then were analyzed by luciferase assay.

As RNF121 seemed to regulate IκBα degradation, a general feature in the process of NF-κB activation [3,7], we then investigated whether RNF121 silencing affects NF-κB activation upon stimulation of innate immunity receptors [15]. We observed that RNF121 silencing also inhibited IκBα degradation and ensuing NF-κB activation following Toll-Like-Receptor 3 (TLR3) stimulation with poly (I:C) (Figure 4A) while it had no effect on the stimulation of the IFNβ promoter (Figure 4B) confirming the specificity of RNF121 in the NF-κB pathway. Similarly, RNF121 knock down impaired NF-κB activation after stimulation of Toll-Like-Receptor 4 (TLR4), retinoic acid-inducible gene 1 (RIG-I), nucleotide-binding oligomerization domain-containing protein 1 (NOD1) and NOD2 with lipopolysaccharide (LPS), viral RNAs, γ-D-Glu-mDAP from peptidoglycan (IE-DAP) or muramyl dipeptide (MDP) respectively (Figure 4C, D, E and F). Finally, in cells exposed to the DNA-damaging agent etoposide, which relies on NEMO SUMOylation and phosphorylation to convey NF-κB activity [16], the transcription factor activation was reduced again (Figure 4G), suggesting that RNF121 acts as a broad regulator of NF-κB signaling.

In summary, we provide evidence that RNF121, a Golgi apparatus-anchored E3 ubiquitin ligase, participates in NF-κB activation. When overexpressed, RNF121 promotes NF-κB activity. While ubiquitination of specific key transmitters is required for the NF-κB signaling [2-5], our data indicate that ubiquitination of RIP1 (Figure 3B and C), IRAK1 or RIP2 (data not shown) following the stimulation of TNFR, TLR4 or NOD1 respectively, was not affected when RNF121 was silenced. Moreover, although the phosphorylation of both IKK and its target IκBα was normal in RNF121 siRNA-transfected cells, IκBα degradation and the resulting p65/p50 NF-κB dimers redistribution were impaired. These observations suggest that RNF121 is involved in the proteasomal degradation of IκBα [7].

Further works are required to delineate the molecular framework employed by RNF121 to regulate IκBα degradation. IκBα degradation involves a K^48^-linked ubiquitination [7] that is mediated by a specific E3 ubiquitin ligase SCF^β-TrCP^ [17-19]. The F-box component of this E3, β-TrCP, recognizes the IκBα degron formed following phosphorylation by IKK and thus couples IκBα phosphorylation to ubiquitination [7]. While endogenous RNF121 and IκBα were found in the same immuno-complex (Figure 3J), RNF121 did not appear to directly ubiquitinate IκBα (Additional file 7A). We then hypothesize that RNF121 controls SCF^β-TrCP^ function on IκBα in a complex through ubiquitination and this aspect merits future exploration. Indeed, the Nedd8 ubiquitin-like molecule regulates the assembly and catalytic activity of the SCF complex [20]. Interestingly, a significant pool of β-TrCP co-localized with the Golgi Apparatus where is anchored RNF121 (Additional file 8) and in preliminary experiments, endogenous RNF121 and β-TrCP were detected in the same complex (data not shown). However, we do not rule out the hypothesis that RNF121 also modulates the ubiquitination of other proteins of the SCF complex as Skp1, Cul1 or Rbx1/Roc1 [7].

In conclusion, over and above its previously known roles, Golgi Apparatus seems to be also involved in NF-κB activation via the E3 ubiquitin ligase RNF121.

## Additional files

Additional file 1:
**Design of the siRNA library.**
Additional file 2:
**Methods description.**
Additional file 3:
**RNF175, a close homolog of RNF121, does not regulate NF-κB activation.**
Additional file 4:
**RNF121 silencing sensitizes cells to TNFα-mediated apoptosis.**
Additional file 5:
**RNF121 silencing delays IκBα degradation.**
Additional file 6:
**A pool of IκBα co-localizes with RNF121.**
Additional file 7:
**RNF121 does not directly ubiquitinate IκBα. (ΠΔΦ 597 κβ)**
Additional file 8:
**Intracellular localization of β-TrCP.**

## Abbreviations

AP-1: Activator protein 1
ERK: Extracellular signal-regulated Kinases
IFN: Interferon
IκBs: Inhibitors of NF-κB
IKK: IκBs kinase
ISRE: IFN stimulated response element
MAPK: Mitogen-activated protein Kinase
NFAT: Nuclear factor of activated T-cells
NEMO: NF-κB essential modulator
NF-κB: Nuclear factor-κB
NLR: NOD-Like Receptor
NOD: Nucleotide-binding oligomerization domain-containing protein
RIG-I: Retinoic acid-inducible gene I
RIP1: Receptor interacting protein 1
RLR: RIG-I-like receptor
RNF121: Ring finger protein 121
TLR: Toll-like receptor
TNFα: Tumor necrosis factor α
TRADD: TNF Receptor type 1-associated death domain protein
TRAF: TNF receptor-associated factor
