# Supplementary material for: The E3 ubiquitin ligase RNF121 is a positive regulator of NF-κB activation
Source: Cell Commun Signal. 2014 Nov 12;12:72. doi: 10.1186/s12964-014-0072-8 (PMC4232610; doi:10.1186/s12964-014-0072-8)
Supplement: Additional file 2: — Methods description. [file 12964_2014_72_MOESM2_ESM.docx]

**Additional File 2: Methods description**

Cell culture and reagents

HEK293T and HeLa cells were provided from the American Type Culture Collection (ATTC). HEK293T cell lines stably expressing NOD1, NOD2, TLR3, or TLR4-MD2-CD14 complex were obtained from InvivoGen (San Diego, CA, USA). All cells were cultured in standard conditions. Depending on the cell line, different ligands were used: TNFα (R&D Systems, Mineapolis, MN, USA) was used at 10 ng.ml^-1^, TLR3 agonist poly (I:C), TLR4 agonist LPS and NODs agonists iE-DAP and Muramyl dipeptide (MDP) were obtained from Invivogen and used at final concentration of 10 μg.ml^-1^. Sendai virus (SeV) used to stimulate the RLRs was kindly provided by Dr D. Garcin (Department of Microbiology and Molecular Medicine, University of Geneva School of Medicine, Geneva, Switzerland). Etoposide (Sigma, St. Louis, MO, USA) was used at the concentration of 40 μM.

siRNA and transfections

siRNA library targeting the 46 E3 ubiquitin ligases (two oligonucleotides per target) with at least a transmembrane domain [1] was purchased from Sigma (Additional File 2). Additional siRNA sequences used were also purchased from Sigma and were:

RNF121a 5'-CCUUCUCAUCUCUGGCCGA[dT][dT]-3'

RNF121b 5'-GCAACUGCUGGACUGGCUU[dT][dT]-3'

RNF175a 5'-GAGAUCUGCUCAGACUACA[dT][dT]-3'

RNF175b 5'-GUUACUUGGCGAUCAUGUU[dT][dT]-3'

RIP1a 5’-CCACUAGUCUGACGGAUAA[dT][dT]-3'

RIP1b 5’-GCAAAGACCUUACGAGAAU[dT][dT]-3'

XIAPa 5’-GGACCAAGACAAGAUUGAA[dT][dT]-3'

XIAPb 5’-CGAGGGCAUAUAUGAAGAA[dT][dT]-3'

HEK293T cells were transfected using the phosphate Calcium precipitation method, whereas HeLa cells transfections were performed using Oligofectamine (Invitrogen, Life Technologies, Grand Island, NY, USA) according to the manufacturer’s instructions. siRNA were used at a final concentration of 20 nM.

DNA transfection

Transfection of HEK293T cells was performed using the calcium phosphate precipitation method. Transfection of HeLa cells by DNA was performed using Lipofectamine 2000 (Invitrogen, Life Technologies,). The plasmid encoding myc-tagged RNF121 was purchased from Origen Technologies (Rockville, MD, USA).

Luciferase essay

Cells were co-transfected with 50 ng of firefly luciferase constructs under the control of the NF- κB or the IFN-β promoter and 10 ng of the renilla luciferase pRL-TK plasmid (Promega, Madison, WI, USA). The transfections were performed using Fugene 6 (Promega) according to the manufacturer’s instructions. The next day, cells were stimulated or not with the indicated ligand for six hours. Transfected cells were collected and luciferase activity was assessed using the Dual-luciferase reporter assay (Promega) on a Fluorostar Optima (BMG Labtech, Ortenberg, Germany). Each experiment was carried out in triplicates. For each sample, to obtain relative fluorescence units (RLU), firefly luciferase fluorescence units were normalized to renilla luciferase fluorescence units.

Protein extraction and immunoblotting analysis

Cells were lysed in lysis buffer (20 mM Tris-HCl (pH 7.4), 137 mM NaCl, 2 mM EDTA, 1% Triton X-100, 2 mM sodium pyrophosphate, 10% glycerol, 25 mM β-glycerophosphate, 1 mM sodium orthovanadate) supplemented with Complete protease inhibitor mixture (Roche Molecular Biochemicals, Meylan, France). 10 nM of calyculin A was added to the lysis buffer when IKK phosphorylation was assessed. After incubation on ice for 10 minutes, lysates were cleared by centrifugation at 10,000 *g* at 4°C and total protein concentration was determinated with a micro BCA kit (Thermo Scientific, Illkirch, France). 10 µg of each lysate were denaturated by boiling for 5 minutes in SDS sample Buffer (Novex, San Diego, CA, USA) plus 10% of β-mercaptoethanol (Sigma) and resolved by SDS-polyacrylamide gel electrophoresis. Immunoblotting analysis was performed with specific antibodies and the Ag-Ab complexes were visualized by chemiluminescence (Immobilon Western, Merck Millipore, Billerica, MA, USA).

Subcellular fractionations

Cells were collected and lysed mechanically with a 27 1⁄2–gauge syringe (BD Biosciences) in lysis buffer H60 (20 mM Hepes (pH 7.9), 1.5 mM MgCl_2_, 60 mM KCl, with protease inhibitors). Samples were centrifuged 5 minutes at 1000 g to remove nuclei and unbroken cells. Heavy membranes (HM) were obtained after a centrifugation at 10,000 g for 10 minutes and cytosolic fractions were collected after centrifugation at 25,000 g for 20 minutes.

Immunofluorescence

Cells were fixed in a solution of 4% paraformaldehyde for 10 minutes, and then permeabilized with 0,15% Triton X-100 in PBS for 15 minutes. Non specific sites were blocked by incubating cells in a solution of 2% BSA in PBS for one hour, then cells were incubated for one hour with the primary antibodies. Three washes for 5 minutes in PBS were performed followed by an incubation for one hour with the specific Alexa Fluor secondary antibodies (InvitroGen). Nuclei were stained with DAPI (InvitroGen) and cells were again washed three times with PBS. Images were acquired with a Leica SP5 confocal microscope (Leica Microsystems, Wetzlar, Germany) equipped with a 63x oil immersion fluorescence objective. When required, the pixel intensity was quantified using Imagej software.

Enzyme-Linked Immuno Sorbent Assay (ELISA)

Cells previously transfected with siRNA were stimulated overnight with TNFα at 1 ng.ml^-1^, then the culture supernatants were collected and IL-8 production was assessed by ELISA assay following the manufacturer’s protocol (Quantikine ELISA, human IL-8, R&D Systems).

Immunoprecipitation

Cells were lysed in lysis buffer (20 mM Tris-HCl (pH 7.4), 137 mM NaCl, 2 mM EDTA, 1% Triton X-100, 2 mM sodium pyrophosphate, 10% glycerol, 25 mM β-glycerophosphate, 1 mM sodium orthovanadate) supplemented with Complete protease inhibitor mixture (Roche Molecular Biochemicals, Meylan, France) for 20 minutes at 4°C. After a centrifugation at 10,000 *g* for 10 minutes, the lysates were precleared with protein G-sepharose beads (Roche Molecular Biochemicals) for 30 minutes, and then incubated with antibody and fresh protein G-sepharose beads for 2 hours at 4°C. The beads were washed four times and immunocomplexes were denaturated and resolved by SDS-PAGE.

Subcellular fractionation for immunoblotting analysis of the nuclear translocation of p65

5-10 x 106 cells were washed with 1 x PBS and incubated for 5 min at 4°C in 200 μl Buffer A (10 mM HEPES pH 7.4, 10 mM KCl, 0.1 mM EDTA, 0.1 mM EGTA, 1 mM DTT, 1 mM Na_3_VO_4_, 1 mM NaF, and Complete protease inhibitor cocktail). Plasma membranes were solubilized by incubation with 12.5 μl of 10% Igepal (Sigma) for 5 min at 4°C. Nuclei were collected by centrifugation at 600 *g* and washed twice with buffer A. Nuclear proteins were then extracted by incubation with 30 μl Buffer B (20 mM HEPES pH 7.4, 400 mM NaCl, 1 mM EDTA, 1 mM EGTA, 1 mM DTT, 1 mM Na3VO4, 1 mM NaF, and Complete protease inhibitor cocktail), with shaking. Nuclear extracts were cleared by centrifugation at 13,000 *g* and analyzed by immunoblotting.

Antibodies

The primary antibodies used for immunoblotting were as follows: Mouse monoclonal anti-TRAF2 (Santa Cruz Biotechnology, Santa Cruz, CA, USA clone F-2; 1:2000 dilution), mouse monoclonal anti-Tubulin (Santa Cruz Biotechnology, clone 10D8; 1:5000), rabbit polyclonal anti-RNF121 (Sigma, HPA046041; 1:2000), mouse monoclonal anti-Myc (Cell Signaling Technology, Danvers, MA, USA, clone 9B11; 1:2000 dilution), rabbit polyclonal anti-Calnexin (BD Biosciences, East Rutherford, NJ, USA; 1:5000), Rabbit polyclonal anti-GAPDH (Sigma; 1:20000), rabbit polyclonal anti-p65 (Santa Cruz Biotechnology, C-20; 1:5000), Mouse monoclonal anti-p50 (Santa Cruz Biotechnology; 1:2000), mouse monoclonal anti-PARP (BD Biosciences, clone C2-10; 1:4000), rabbit polyclonal anti-ERK1/2 (Cell Signaling Technology, clone 9102; 1:5000), rabbit polyclonal anti-phosphorylated ERK1/2 (Cell Signaling Technology, clone E10; 1:5000), mouse monoclonal anti-phosphorylated-IκBα (Cell Signaling Technology, clone 5A5; 1:2000), rabbit polyclonal anti-IκBα (Cell Signaling Technology; 1:5000), mouse monoclonal anti- IκBα (Santa Cruz Biotechnology, clone H4; 1:2000), rabbit polyclonal anti-phosphorylated IKKα/β (Cell Signaling Technology, clone 16A6; 1:1000), mouse monoclonal anti-RIP1 (BD Biosciences, clone 38/RIP; 1:4000), mouse monoclonal anti-TNFR1 (Santa Cruz Biotechnology, clone H-5; 1:2000) and rabbit polyclonal anti-β-TRCP/HOS (Santa Cruz Biotechnology, H-300; 1:1000). The primary antibodies used for immunofluorescence microscopy were: Mouse monoclonal anti-Myc (Cell Signaling Technology, clone 9B11; 1:400), mouse monoclonal anti-GM130 (BD Biosciences, 1:400), rabbit polyclonal anti-RNF121 (Sigma, HPA046041; 1:400), rabbit polyclonal anti-p65 (Santa Cruz Biotechnology, C-20; 1:500), rabbit polyclonal anti-β-TRCP/HOS (Santa Cruz Biotechnology, H-300; 1:200) and mouse monoclonal anti-IκBα (Cell Signaling Technology; clone L35A5; 1:200). The primary antibodies used for immunoprecipitation were a mouse monoclonal TNFR1 (Santa Cruz Biotechnology, clone H-5), and a rabbit polyclonal anti-RNF121 obtained after a rabbit immunization with the following peptide (CKEKVDLKRMFSNPWERPHV) and followed by a purification (Biotem, Apprieu, France).

Statistical analysis

Two-way ANOVA tests with *post hoc* Tukey’s analysis were used to assess the statistical significance of differences (Prism GraphPad Software), and *P* values are indicated in the figure legends. In some cases, as indicated, data were also compared in Student’s *t*-tests. Differences were considered to be significant if *P* < 0.05. *****P*<0.0001, ****P* < 0.001, **0.001 <*P* < 0.01, *0.01 <*P* < 0.05. ns, not significant. The data shown in each histogram are the means ± SD from three independent experiments.

Additional Reference

1. Li W, Bengtson MH, Ulbrich A, Matsuda A, Reddy VA, Orth A, Chanda SK, Batalov S, Joazeiro CA: **Genome-wide and functional annotation of human E3 ubiquitin ligases identifies MULAN, a mitochondrial E3 that regulates the organelle's dynamics and signaling.** *PLoS One* 2008, **3:**e1487.
