# Supplementary material for: The E3 ubiquitin ligase RNF121 is a positive regulator of NF-κB activation
Source: Cell Commun Signal. 2014 Nov 12;12:72. doi: 10.1186/s12964-014-0072-8 (PMC4232610; doi:10.1186/s12964-014-0072-8)
Supplement: Additional file 3: — RNF175, a close homolog of RNF121, does not regulate NF-κB activation. [file 12964_2014_72_MOESM3_ESM.pdf]

A

```
RNF121          MAAVVEVEVGGAAGER--ELDEVDMSDLSPEEQWRVEHARMHAKHRGHEAMHAEMVLILI 58
RNF175          MAAGTAARKAAPVLEAPPQQEQLSHTKLSAEDTWNLQQERMYKMHRGHDSMHVEMILIFL 60
                *** . .. .... *   : :::. :.**.*: *.::: **:  ****::**.**:**::

RNF121          ATLVVAQLLLVQWKQRHPRSYNMVTLFQMWVVPLYFTVKLHWWRFLVIWILFSAVTAFVT 118
RNF175          CVLVIAQIVLVQWRQRHGRSYNLVTLLQMWVVPLYFTIKLYWWRFLSMWGMFSVITSYIL 120
                ..**:**::****:*** ****:***:*********:**:****** :* :**.:*:::

RNF121          FRATRKPLVQTTPRLVYKWFLLIYKISYATGIVGYMAVMFTLFGLNLLFKIKPEDAMDFG 178
RNF175          FRATRKPLSGRTPRLVYKWFLLIYKLSYAFGVVGYLAIMFTMCGFNLFFKIKARDSMDFG 180
                ********   **************:*** *:***:*:***: *:**:****..*:****

RNF121          ISLLFYGLYYGVLERDFAEMCADYMASTIGFYSESGMPTKHLSDSVCAVCGQQIFVDVSE 238
RNF175          IVSLFYGLYYGVMGRDFAEICSDYMASTIGFYSVSRLPTRSLSDNICAVCGQKIIVELDE 240
                *  *********: *****:*:*********** * :**: ***.:******:*:*::.*

RNF121          EGIIENTYRLSCNHVFHEFCIRGWCIVGKKQTCPYCKEKVDLKRMFSNPWERPHVMYGQL 298
RNF175          EGLIENTYQLSCNHVFHEFCIRGWCIVGKKQTCPYCKEKVDLKRMISNPWERTHFLYGQI 300
                **:*****:************************************:******.*.:***:

RNF121          LDWLRYLVAWQPVIIGVVQGINYILGLD 326
RNF175          LDWLRYLVAWQPVVIGIVQGINYSLGLE 328
                *************:**:****** ***:
```

B

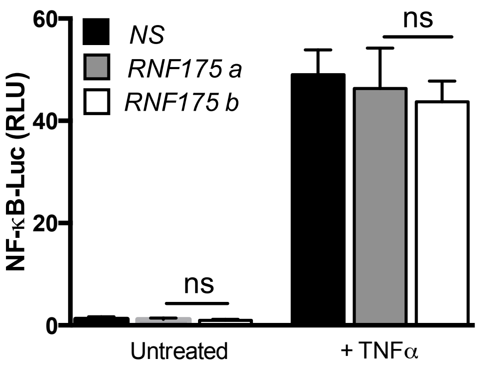

Additional File 3

Additional file 3. RNF175, a close homolog of RNF121, does not regulate NF-κB activation.
(A) CLUSTAL-W was used to generate an alignment of RNF121 with RNF175. **(B)** HEK293T cells transfected with a control non-specific (*NS*) siRNA or with siRNAs against RNF175 (*RNF175 a* or *b*), were also transfected 48 hrs later with an NF-κB reporter. 24 hrs later, the cells were either left unstimulated or were stimulated with TNFα (10 ng/ml) for 6 hrs and then were analyzed by luciferase assay. The results were normalized against *Renilla* luciferase activity. The data shown are the means ± SD from three independent experiments. ns: not significant. RLU, Relative Light Units.
