# Supplementary material for: The E3 ubiquitin ligase RNF121 is a positive regulator of NF-κB activation
Source: Cell Commun Signal. 2014 Nov 12;12:72. doi: 10.1186/s12964-014-0072-8 (PMC4232610; doi:10.1186/s12964-014-0072-8)
Supplement: Additional file 4: — RNF121 silencing sensitizes cells to TNFα-mediated apoptosis. [file 12964_2014_72_MOESM4_ESM.pdf]

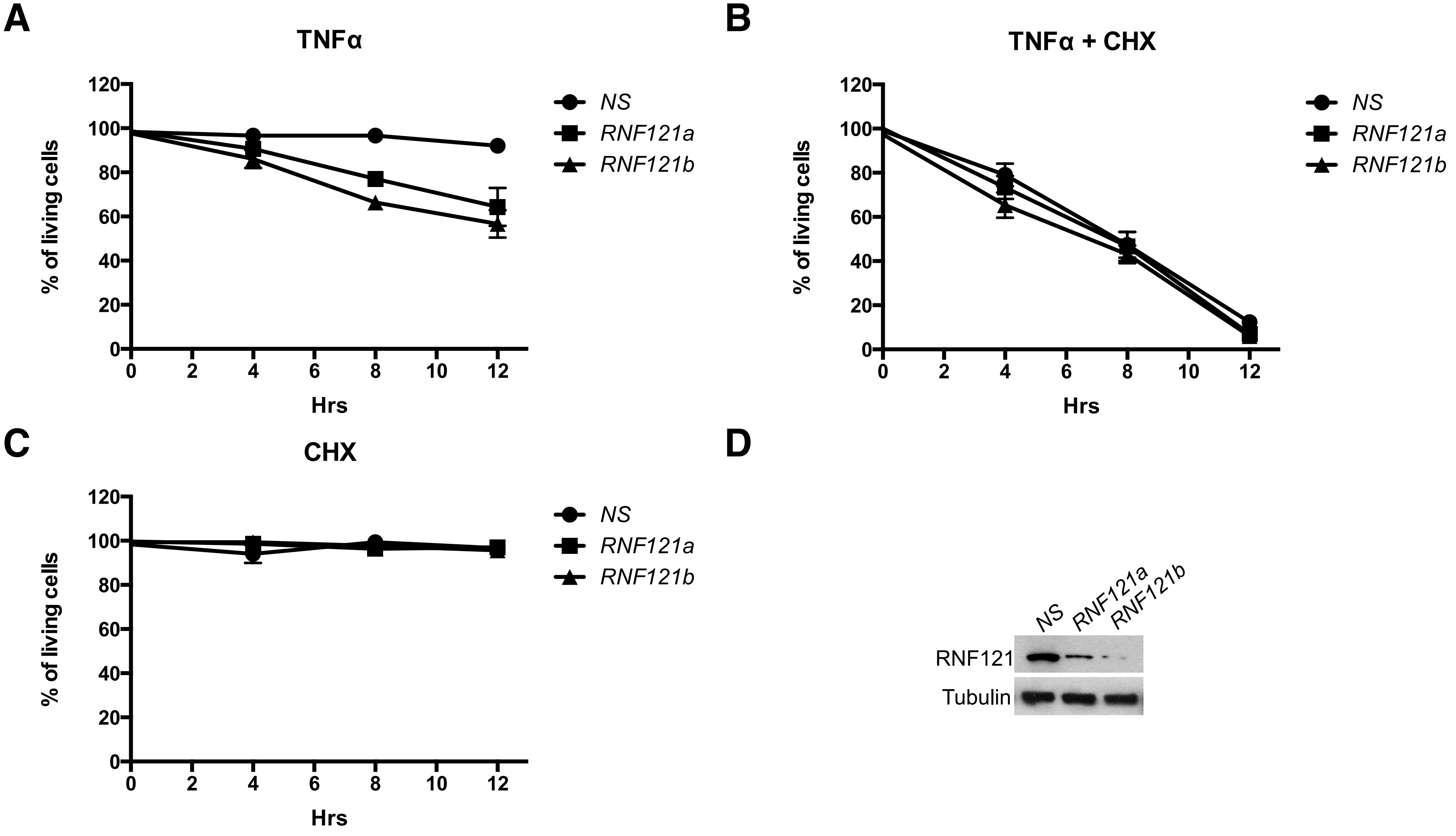

Additional File 4

Additional file 4. RNF121 silencing sensitizes cells to TNFα-mediated apoptosis.
HeLa cells were transfected with a control non-specific (*NS*) siRNA or with siRNAs against RNF121 (*RNF121 a* or *b*). 72 hrs later, cells were exposed to either TNFα (25 ng/ml) in the absence **(A)** or the presence of CHX (10 μg/ml) **(B)**, or CHX alone **(C)** for the indicated periods of time. The percentage of living cells was measured by trypan blue exclusion. The data shown are the means ± SD from three independent experiments, with 300 cells per condition. The knock down of RNF121 is shown in **(D)**.
