# Supplementary material for: The E3 ubiquitin ligase RNF121 is a positive regulator of NF-κB activation
Source: Cell Commun Signal. 2014 Nov 12;12:72. doi: 10.1186/s12964-014-0072-8 (PMC4232610; doi:10.1186/s12964-014-0072-8)
Supplement: Additional file 5: — RNF121 silencing delays IκBα degradation. [file 12964_2014_72_MOESM5_ESM.pdf]

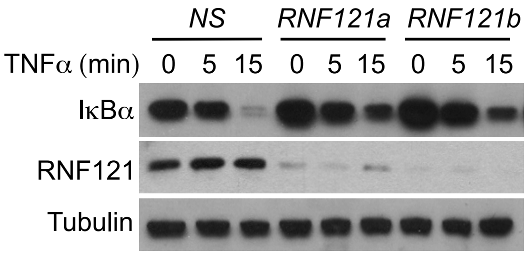

Additional File 5

Additional file 5. RNF121 silencing delays IκBα degradation.
HeLa cells transfected with a *NS* siRNA or *RNF121*-specific siRNAs were stimulated 72 hrs later with TNFα (10 ng/ml) for the indicated times. Cell extracts were then analyzed by immunoblotting with antibodies specific for the indicated proteins.
