# Supplementary material for: The E3 ubiquitin ligase RNF121 is a positive regulator of NF-κB activation
Source: Cell Commun Signal. 2014 Nov 12;12:72. doi: 10.1186/s12964-014-0072-8 (PMC4232610; doi:10.1186/s12964-014-0072-8)
Supplement: Additional file 6: — A pool of IκBα co-localizes with RNF121. [file 12964_2014_72_MOESM6_ESM.pdf]

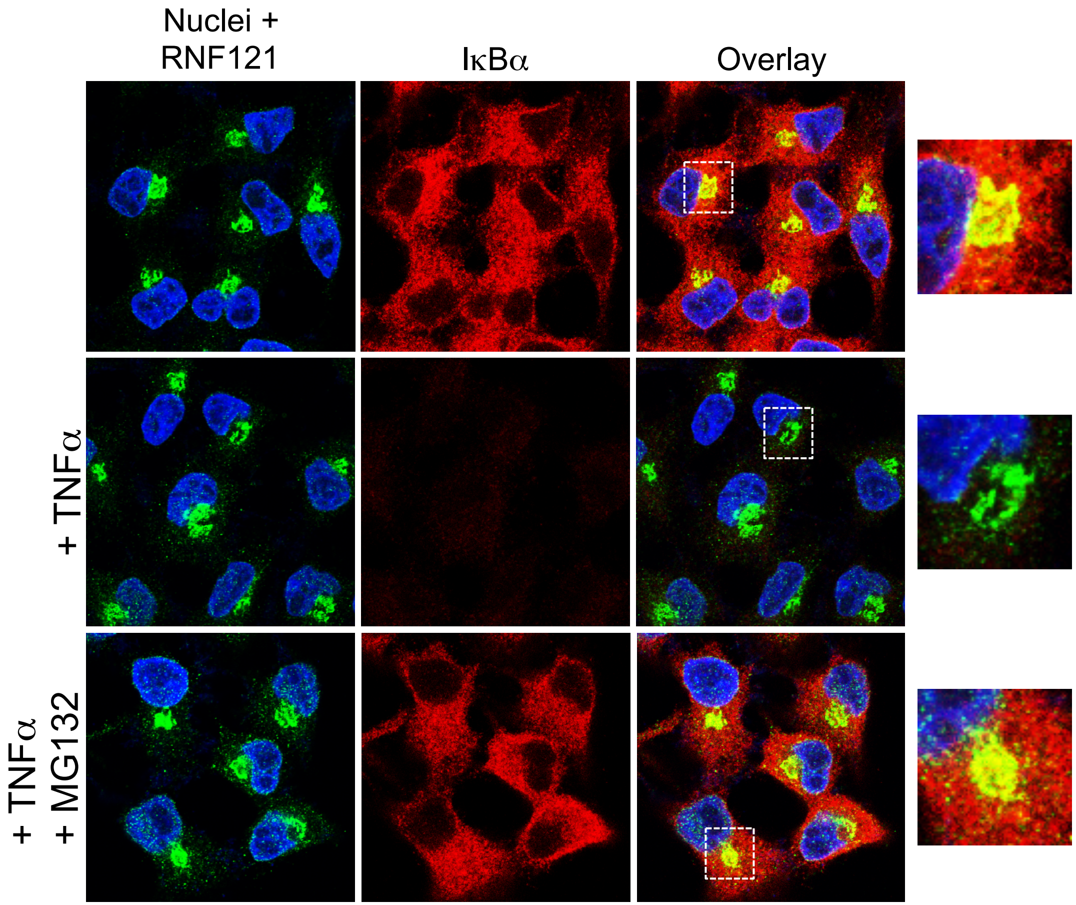

Additional File 6

Additional file 6. A pool of IκBα co-localizes with RNF121

(A) HeLa cells were treated or not with TNFα (10 ng/ml) for 15 min in the absence or the presence of the proteasome inhibitor MG132 (10 μM), then the localization of RNF121 and IκBα were investigated by immuno-fluorescence. Nuclei were stained with DAPI. Representative images are shown, with the boxed areas enlarged on the right.
