# Supplementary material for: The E3 ubiquitin ligase RNF121 is a positive regulator of NF-κB activation
Source: Cell Commun Signal. 2014 Nov 12;12:72. doi: 10.1186/s12964-014-0072-8 (PMC4232610; doi:10.1186/s12964-014-0072-8)
Supplement: Additional file 7: — RNF121 does not directly ubiquitinate IκBα. (ΠΔΦ 597 κβ) [file 12964_2014_72_MOESM7_ESM.pdf]

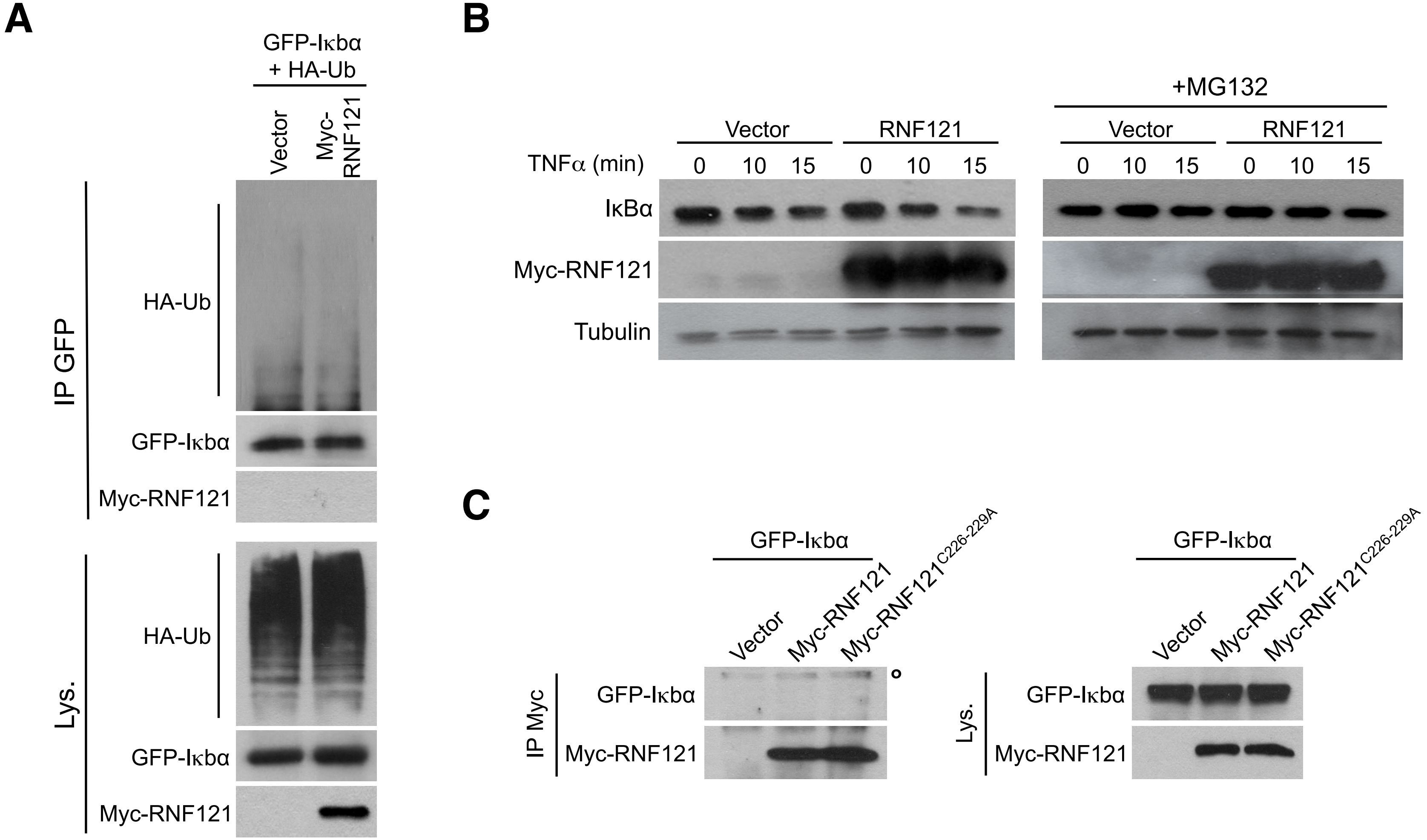

Additional File 7

Additional file 7. RNF121 does not directly ubiquitinate IκBα.

(A) HEK293 cells were transfected with a plasmid encoding myc-tagged RNF121 or with an empty vector as a control and with plasmids encoding GFP-tagged IκBα and HA-tagged ubiquitin (Ub). Twenty-four hours after transfection, IκBα was immunoprecipitated with an anti-GFP and its ubiquitination was assessed with an anti-HA (upper panel). Expression of the proteins was examined by immunoblotting with the indicated antibodies in the lysat (Lys.) (lower panel). **(B)** HEK293 cells were transfected with a plasmid encoding myc-tagged RNF121 or with an empty vector as a control. 24 hrs later, cells were stimulated with TNFα (10 ng/ml) for the indicated times in the presence or the absence of the proteasome inhibitor MG132 (10 μM) and cell extracts were analyzed by immunoblotting as indicated. **(C)** HEK293 cells were transfected with a plasmid encoding myc-tagged RNF121 or with an empty vector as a control and with a plasmid encoding GFP-tagged IκBα. Twenty-four hours after transfection, myc-RNF121 was immunoprecipitated with an anti-myc antibody. Samples were then analyzed by immunoblotting. ° indicates a non specific band.
