# Supplementary material for: The E3 ubiquitin ligase RNF121 is a positive regulator of NF-κB activation
Source: Cell Commun Signal. 2014 Nov 12;12:72. doi: 10.1186/s12964-014-0072-8 (PMC4232610; doi:10.1186/s12964-014-0072-8)
Supplement: Additional file 8: — Intracellular localization of β-TrCP. [file 12964_2014_72_MOESM8_ESM.pdf]

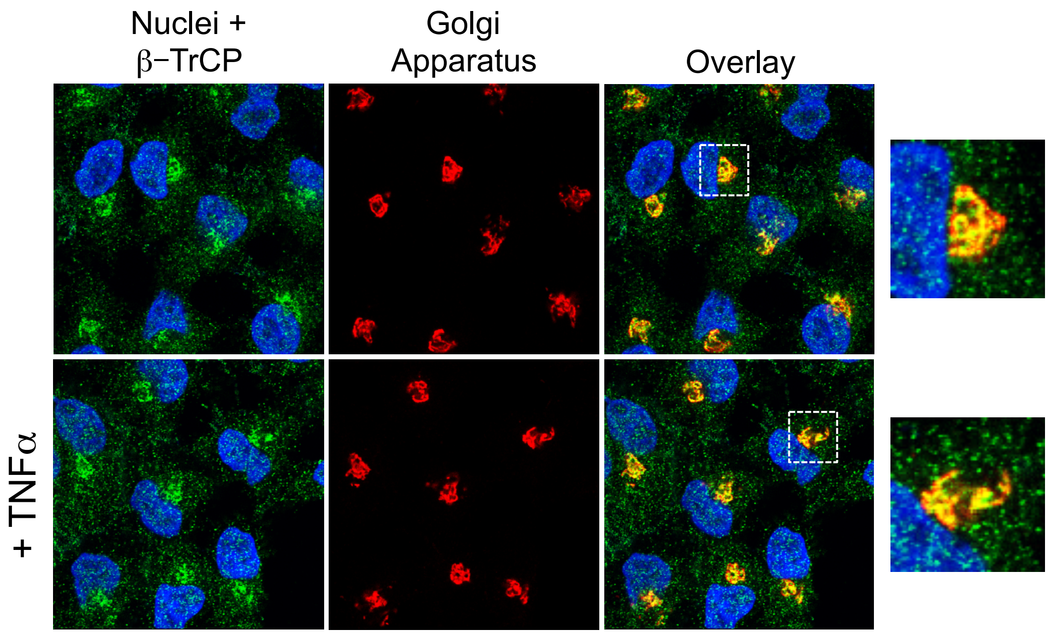

Additional File 8

Additional file 8. Intracellular localization of β-TrCP.
HeLa cells were treated or not with TNFα (10 ng/ml) for 15 min, then β-TrCP localization was investigated by immuno-fluorescence. GM130 was used as a marker for the Golgi Apparatus. Nuclei were stained with DAPI. Representative images are shown, with the boxed areas enlarged on the right.
